# In Situ Decoration of Gold Nanoparticles on Graphene Oxide via Nanosecond Laser Ablation for Remarkable Chemical Sensing and Catalysis

**DOI:** 10.3390/nano9091201

**Published:** 2019-08-26

**Authors:** Parvathy Nancy, Anju K Nair, Rodolphe Antoine, Sabu Thomas, Nandakumar Kalarikkal

**Affiliations:** 1 School of Pure and Applied Physics, Mahatma Gandhi University, Kottayam 686560, India; 2 Department of Physics, St. Teresas’s College, Ernamkulam 682011, India; 3 Institut Lumière Matière, UMR 5306 CNRS, Université Claude Bernard Lyon 1, Domaine Scientifique de La Doua, Batiment Kastler, 10 rue Ada Byron, 69622 Villeurbanne CEDEX, France; 4 International and Inter University Centre for Nanoscience and Nanotechnology, Mahatma Gandhi University, Kottayam 686560, India; 5 School of Chemical Sciences, Mahatma Gandhi University, Kottayam 686560, India

**Keywords:** gold nanoparticles, graphene oxide, laser ablation, Au-GO nano-hybrid

## Abstract

Gold decorated graphene-based nano-hybrids find extensive research interest due to their enhanced chemical catalytic performance and biochemical sensing. The unique physicochemical properties and the very large surface area makes them propitious platform for the rapid buildouts of science and technology. Graphene serves as an outstanding matrix for anchoring numerous nanomaterials because of its atomically thin 2D morphological features. Herein, we have designed a metal-graphene nano-hybrid through pulsed laser ablation. Commercially available graphite powder was employed for the preparation of graphene oxide (GO) using modified Hummers’ method. A solid, thin gold (Au) foil was ablated in an aqueous suspension of GO using second harmonic wavelength (532 nm) of the Nd:YAG laser for immediate generation of the Au-GO nano-hybrid. The synthesis strategy employed here does not entail any detrimental chemical reagents and hence avoids the inclusion of reagent byproducts to the reaction mixture, toxicity, and environmental or chemical contamination. Optical and morphological characterizations were performed to substantiate the successful anchoring of Au nanoparticles (Au NPs) on the GO sheets. Remarkably, these photon-generated nano-hybrids can act as an excellent surface enhanced Raman spectroscopy (SERS) platform for the sensing/detection of the 4-mercaptobenzoic acid (4-MBA) with a very low detection limit of 1 × 10^−12^ M and preserves better reproducibility also. In addition, these hybrid materials were found to act as an effective catalyst for the reduction of 4-nitrophenol (4-NP). Thus, this is a rapid, mild, efficient and green synthesis approach for the fabrication of active organometallic sensors and catalysts.

## 1. Introduction

Nano sized particles of noble metals, particularly gold NPs (Au NPs), have ample scope in research due to their unique optical, electrical and catalytic properties and their potential applications in material science, physics, chemistry and in many interdisciplinary fields [1,2,3]. The Au NPs have the tendency of aggregation when they are in a solution on account of their superior surface energy, which results in a reduced catalytic activity and stability [4]. This disadvantage can be solved by anchoring Au NPs on various matrices such as SiO_2_, polymer and carbon microspheres, etc. [5,6,7]. Among these matrices, graphene derivatives like graphene oxide (GO), and reduced graphene oxide (rGO) have been subjected to vast study owing to an excessive surface area, planar structure, and exceptional physicochemical properties [8]. GO is a better choice when compared with graphene in terms of a low production cost, high yield manner and more application suitability. Having a rich O_2_ containing functional groups on its edges and basal planes make GO hydrophilic and a good support for the surface functionalization and nanoparticle immobilization. Until now, extensive research has been done on the development of GO or rGO supported Au NPs for applications in the field of catalysts, sensors and medicine. Au NPs and GO nano-hybrids shows novel and increased physicochemical properties, which is considered to be accomplished from the strong interaction between these two constituents [9,10,11].

Graphene sheets embellished with metal nanoparticles are brilliant materials having potential use in energy storage, optoelectronics, bio sensors or catalysts, because of their unique physical and chemical characteristics [12,13]. The surface plasmon resonance of the metallic nanoparticles, interaction of graphene sheets with light, formation of bio sensors by means of bio conjugation, inflation of catalytic activity of nanoparticles [14,15], etc. are controlled by the unique conductivity and volatile features of graphene and metal nanoparticles, respectively. Usually, the graphene- metal nano-hybrids are fabricated by the synchronized reduction of metal salts and graphene derivatives. In such methods, surfactants or co-ligands on the synthesized nanoparticles are utilized to augment stability and to reduce aggregation behavior. At the same time, impurities are also produced along with the entire procedure and final products [16,17]. Generally, the metal nanoparticles are capped with ligands in the surface, which foster a cross-chemical effect. As a solution to this dilemma, ligand free metal nanoparticles have been synthesized through alternative top-end and easy pathways, functioning on the basis of principles of green protocols [18,19]. Pulsed laser ablation in liquids (PLAL) is such a method, in which a solid target immersed in a colloid or liquid is irradiated with a suitable pulsed laser beam [20,21,22,23]. Here in PLAL, the possible synergy between the electromagnetic field of the laser pulses and the atoms on the material surface leads to the removal of electrons from the surface, culminating in creation of an electronic cloud and an electron deficiency localized area on the surface of the target. The electronic cloud in turn will attract the surface ions by means of electromagnetic force, resulting in the formation of clusters of atoms and ionized atoms. A cavitation bubble formation is the next process in line, which cages the material that forms crystalline nanoparticles and finally discharges the nanoparticles in to the liquid environment as the bubble blots out [24,25]. The ligand free Au NPs shows better surface activity due to the lack of ligands on its surface so that it can retain on the GO surface by the combined effect of three distinct reasons (a) interplay among the d-orbitals of metal nanoparticles and the pi-electron aromatic network of graphene (b) the strong electrostatic interaction between metal nanoparticles and graphene promotes the effective anchoring of AuNPs on GO (c) desirable distribution of nanoparticles at the defects/wrinkles found in the graphene surface [26,27]. There are reports of synthesis of ligand free Au NPs by PLAL and subsequent blending with graphene with the aid of a variety of lasers [28,29,30]. The prospect of direct synthesis of particles from graphene sheets in liquid medium signifies that the electromagnetic effects from the laser radiation causing some reactive process in GO may govern the reduction process of graphene oxide. Thus, the laser irradiation eliminates the O_2_ functional groups from the graphene sheet, whilst possessing the large inter layer spacing, that characterizes the electronic decoupling of the individual layer in the material and this decoupling effect may give rise to superconductivity effects sometimes [31,32]. In this work, we focus on the decoration of graphene oxide sheets with Au NPs in a novel single step approach. The impact given by a nanosecond radiation-based technique upon the control of synthesis of highly pure Au NPs in a liquid ambience containing graphene oxide layers has been studied. Apart from the conventional synthesis methods and the previously reported synthesis via PLAL, we present the fabrication of the Au-GO hybrid material, especially in the nanosecond regime for the first time. The in situ generation and decoration of the Au NPs on the GO layers are established by photons and the purity, structure, size and stability of the AuNPs were fine-tuned by different laser parameters like laser fluence, repletion rate, laser wavelength, irradiation time, etc. To establish the hypothesis, Au NPs were synthesized in a homogenous aqueous suspension of graphene oxide layers. Moreover, this methodology has proved that it is an impressive protocol to derive graphene oxide-metal nanoparticle assemblies with a potential to be used in a verity of applications where undesirable effects of harmful chemicals can be avoided successfully.

## 2. Experimental Procedure

In order to generate the Au-GO nano-hybrid, a thin gold foil of thickness of 0.1 mm (SIGMA ALDRICH, Missouri, MO, USA, pure trace metal, 99.99%) was properly fixed in the inner side of a glass cuvette, which is filled with 30 mL of an aqueous suspension of the GO solution. The second harmonic beam of an Nd:YAG laser (Litron LPY 674G-10) having a pulse width of 8 ns was focused on to that gold foil with a bi-convex lens having focal length 15 cm at a 10 Hz repletion rate. The spot diameter of the beam was found to be 0.01 mm. Thus, the Au NPs were born and anchored in situ on to the GO single layers through the nanosecond laser radiation strategy technically known as PLAL. GO was prepared previously from the standard graphite powder using a modified Hummers’ method [33]. The whole experiment was performed completely in room temperature.

**Figure 1:**
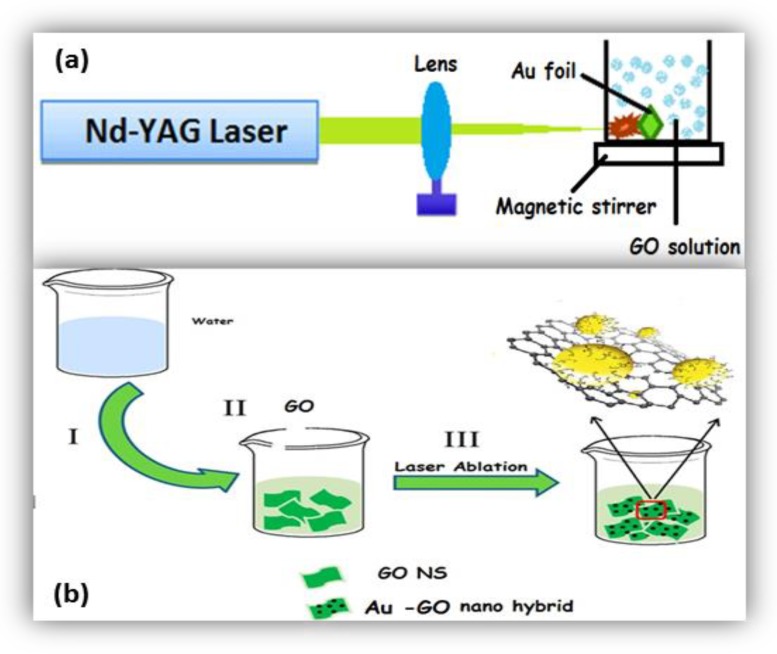
(**a**) The experimental framework of fabrication of Au-GO nano-hybrid; (**b**) synthesis procedure for the Au-GO nano-hybrid.

Figure 1 depicts the experimental arrangement of laser ablation. The Au target is ablated for fifteen minutes (15 min) at four distinct laser energy densities such as 5.3 Jcm^−2^, 7.9 Jcm^−2^, 10.5 Jcm^−2^, and 13.2 Jcm^−2^. The GO solution was kept under stirring with the aid of a magnetic stirrer for uniform distribution/decoration of the Au NPs on the GO matrix.

## 3. Results and Discussion

Here, we focus on the facile generation of the Au-GO nano-hybrid using a laser ablation in nanoseconds. The influence of the nanosecond radiation on the controlled synthesis of uniformly dispensed Au NPs in the aqueous suspension of GO sheets and its applications in chemical sensing and catalysis has been studied. With this focus, Au NPs were produced in situ and were anchored on to single layered graphene-oxide sheets. Our results demonstrate that the growth of ligand-free metal nanoparticles on graphene oxide sheets in a single reaction procedure using a laser ablation is a highly beneficial technique for fast, simple and environmentally friendly synthesis of nanoparticles, hybrids and composites.

### 3.1. UV-Vis Absorption Spectroscopy

**Figure 2:**
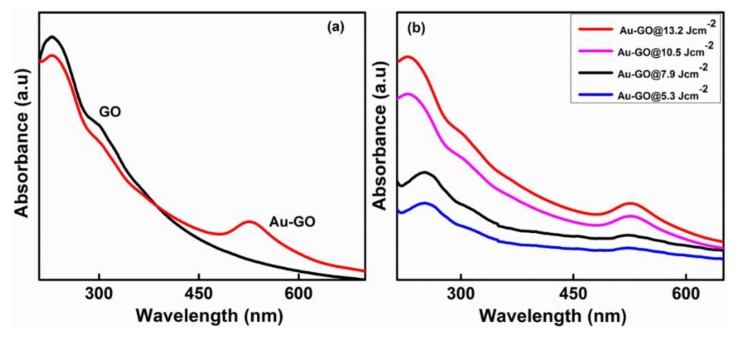
UV-Vis absorption spectrum. (**a**) GO and Au-GO nano-hybrid; (**b**) Au-GO at different laser fluences.

Figure 2a represents the absorption spectrum of a pure GO sheet. The GO sheets display an absorption at 234 nm, which is attributed to the C–C bond’s π ⟶ π* interaction; the tiny shoulder peak at 304 nm is attributed to the C=O bond’s n ⟶ π* interaction. Figure 2b represents the absorption spectra of the Au-GO nano-hybrid. After the ablation process at four distinct laser fluences (5.3 Jcm^−2^, 7.9 Jcm^−2^, 10.5 Jcm^−2^, and 13.2 Jcm^−2^), the absorption peak of the GO in all prepared samples slowly shifted to 255 nm, which implies that a few GO sheets have undergone a reduction due to the interactivity of the GO with a high energy laser beam (Figure 2b). Together with this, the Au-GO nano-hybrid material evince a wide absorption peak at ~526 nm. This is the contribution by the surface plasmon resonance (SPR) of the Au NPs [34,35]. These results indicate that the Au NPs were generated and successfully anchored on the GO matrix. The results also elucidate that the laser fluence is a key-regulating parameter and has a vital role in the size and concentration of Au NPs on the GO matrix.

### 3.2. XPS and Raman Analysis

**Figure 3:**
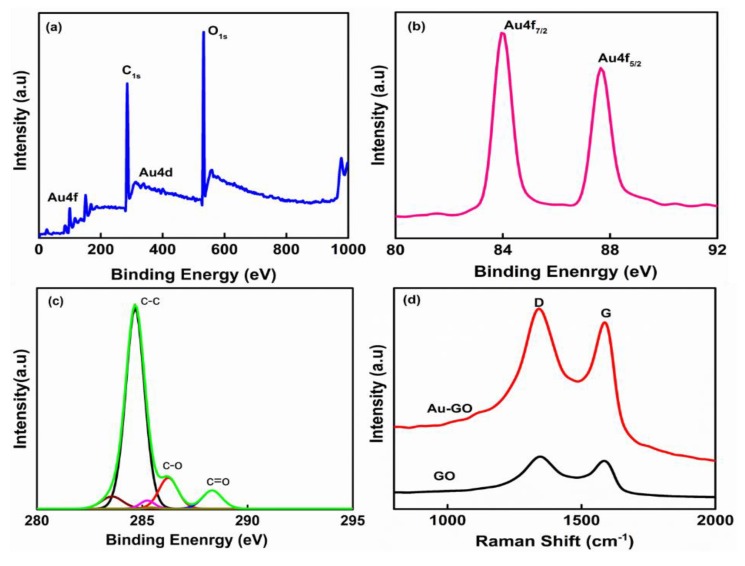
X-ray photoelectron spectroscopy (XPS) and Raman spectra of the Au-GO nano-hybrid. (**a**) Wide scan spectra; (**b**) Au4f spectrum; (**c**) C1s spectrum; (**d**) Raman spectra of the Au-GO and GO.

The wide-scan XPS spectra of the Au-GO nano-hybrid shown in Figure 3a upholds the existence elements of Au, C and O. In order to examine the oxidation states of Au and GO, the X-ray photoelectron spectroscopy (XPS) was employed. Figure 3a depicts the wide scan spectra of the Au-GO hybrid material. In fact, the high-resolution XPS peaks of the deconvoluted C1s spectrum of the Au-GO nano-hybrid shows three different peaks due to the existence of the C=O (288.2 eV), C–O (286.6 eV) and C–C (286.4 eV) functional groups (Figure 3c), while the binding energy at 84 eV and 87 eV were attributed to the Au4f_7/2_ and Au4f_5/2_ peaks (Figure 3b). This result confirms the effective formation of the Au layer over GO.

The Raman spectroscopy was employed additionally to explore the structure of the nano-hybrid and the interactivity between the Au nanoparticles and GO sheets. Here, in the Raman spectra Figure 3d reveals an intense D peak at 1349 cm^−1^ and a G peak at 1580 cm^−1^, which affirms the genesis of a few-layered GO with its graphitic structure. The occurrence of an intense D band at 1349 cm^−1^ in the GO sample clearly indicates the existence of defect sites in the graphene layers. These defects are often assigned to the oxidation of graphite and doping effects in the hexagonal carbon lattice. Furthermore, the I_D_/I_G_ ration (I_D_/I_G_ = 1.09) for the Au-GO hybrid is large compared with the bare GO (I_D_/I_G_ = 0.89); it substantiates the existence of several defects in the Au-GO created during the laser ablation. The evident spike in the I_D_/I_G_ value from GO to Au-GO indicates the successful anchoring of Au on the GO network. Moreover, the nano-hybrids exhibit increased Raman intensity, which was because of the effect of the localized electromagnetic field of the Au nanoparticles and thus it can be a competent substrate for SERS detection.

### 3.3. Morphological Analysis

The surface morphology and size of the Au-GO nano-hybrids were studied using a transmission electron microscopy (TEM) and field emission scanning electron microscopy (FESEM).

**Figure 4:**
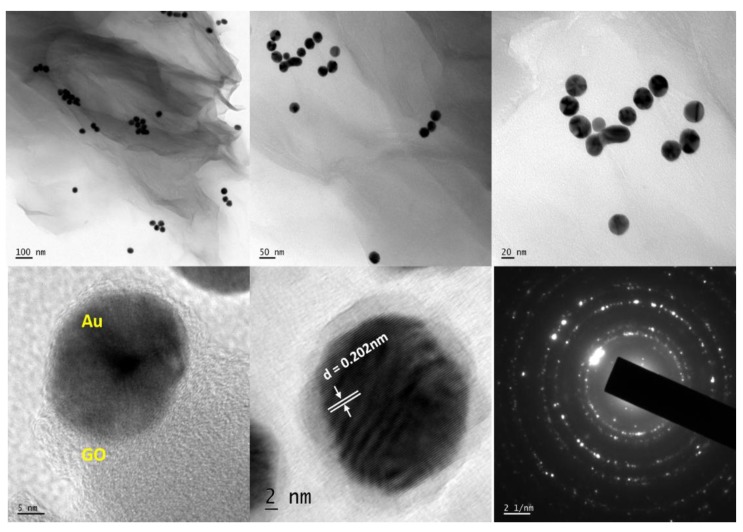
TEM and HRTEM images of the Au-GO nano-hybrid.

**Figure 5:**
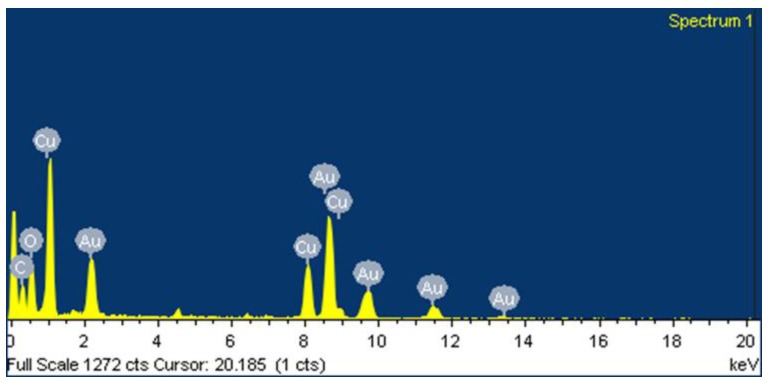
TEM-EDS of the Au-GO nano-hybrid.

The TEM image (Figure 4) of the Au-GO nano-hybrid shows that the small Au nanoparticles of a diameter around ~30 nm are anchored on to the surface of the GO. The HRTEM analysis shows that the synthesized Au-GO material is unbound of any other chemical impurities and elements. The high crystalline nature of the material was clear from the lattice fringes. It also provides clear details regarding the d spacing of the Au nanoparticles. The Au nanoparticles exhibit a darker contrast with an interlayer spacing of 0.202 nm that corresponds to the d-spacing of a (200) plane of Au. In addition, the transmission electron microscope-energy dispersive spectra (TEM-EDS) confirms the presence of highly-pure gold nanoparticles decorated over the GO matrix; it is shown in Figure 5. The EDS reveals that, the most evident intensity peaks are Au, C and O corresponding to the Au NPs and GO sheets.

**Figure 6:**
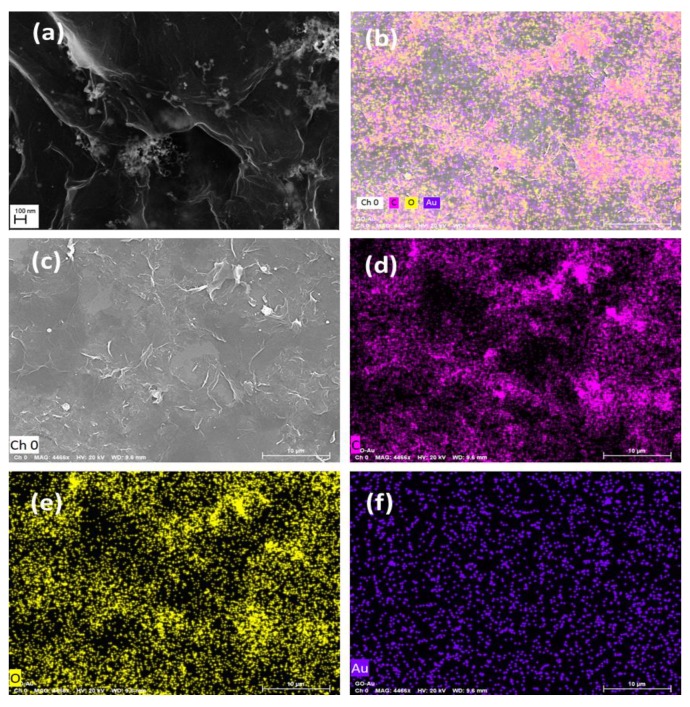
(**a**) Field emission (FESEM) image of the Au-GO nano-hybrid (**b**–**f**) elemental mapping of Au-GO nano-hybrid, where C, O and Au are mapped in pink, yellow and blue colours respectively.

Also, Figure 6 shows the FESEM image and the elemental mapping of the Au-GO nano-hybrid in which the elements Au, C and O are mapped in blue, pink and yellow colour, respectively; the mapping clearly suggests the co-existence of Au and GO in the laser generated nano-hybrid. Figure 6a clearly depicts that the number density of the Au NPs are very high in the vicinity of wrinkles in the GO sheets. These are one of the main reasons for the adsorption of the AuNPs on the GO. The wrinkles present in the GO corresponds to the defect sites and it facilitates the high rate of accumulation and immobilization of the Au NPs on such areas.

### 3.4. SERS Activity of 4-Mercaptobenzoic Acid (4-MBA)

For the SERS measurement, 10 µL of the samples were coated on a silicon wafer (~1 cm^2^). The 4-mercaptobenzoic acid solution (4-MBA solution, 10 μM concentration, and 10 µL quantity) was prepared and drop casted on a silicon wafer and then dried in room temperature. These stock solutions were gradually diluted with de-ionized water to obtain various concentrations of analytes. A confocal Raman spectrometer (WITec alpha 300RA) with a 532 nm excitation wavelength was used for SERS measurements of hence prepared samples. The SERS data were collected over the span of 200–2000 cm^−1^ with 10 s as an integration time.

**Figure 7:**
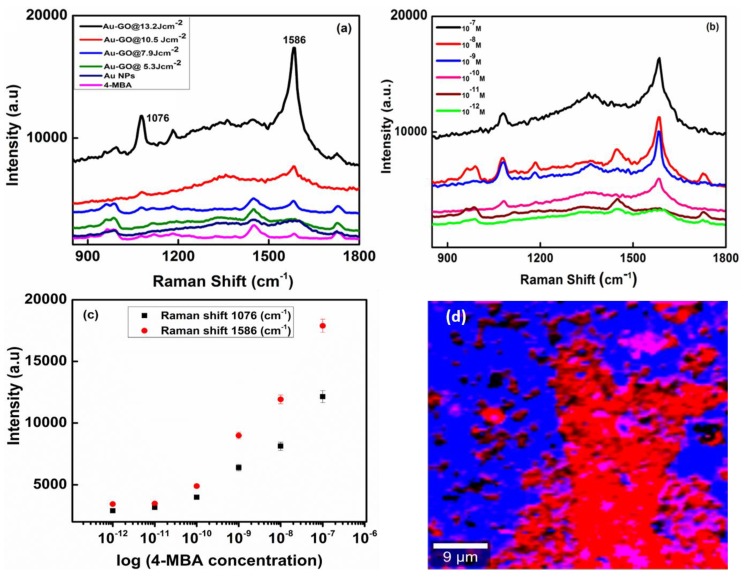
(**a**) The surface enhanced Raman spectroscopy (SERS) spectra of the samples at 10 μM 4-MBA; (**b**) the SERS spectra of Au-GO at different concentrations of 4-MBA; (**c**) calibration data with respect to the average SERS intensities of the peaks of 4-MBA at 1076 cm^−1^ and 1586 cm^−1^ with the 4-MBA concentrations; (**d**) the Raman image of the Au-GO, the red region corresponds to the ‘hot spots’.

The relative SERS enhancement capabilities of the Au-GO nano-hybrids were measured using 4-mercaptobenzoic acid (4-MBA) as the probe molecule, which is highly Raman active and adsorbed on the surface of these hybrid nanostructures. For comparison, Au NPs are also prepared to confirm the SERS activity of the 4-MBA molecules. The 4-MBA is an organic molecule with a thiol group and a carboxylic acid group on the two ends that usually exhibits a strong chemical coupling with metallic surfaces [36]. It is well known that metal nanoparticles incorporated nanostructures give higher SERS enhancement than the bare metal nanoparticles. Metal nanoparticles usually contribute an electromagnetic field enhancement to the SERS signals. Figure 7a represents the SERS spectra of the Au-GO synthesized at different laser energies, capped with 4-MBA (10 µM). In Figure 7a, the SERS spectra exhibit two prominent Raman peaks at 1076 cm^−1^ and 1586 cm^−1^ that is attributed to the ν(C–C) breathing modes of a benzene ring. The other weak bands, 1412 cm^−1^ emerged from the stretching mode of ν_s_ (COO–) and the band at 1182 cm^−1^ from the C–H deformation modes; they were found to be consistent with the previous report [37]. The ν(C–C) mode depicts a dominant peak intensity than the other modes of vibration due to the enhanced coupling between the transition dipole moment and local electric field [38,39]. The samples at 13.2 Jcm^−2^ laser energy displayed more SERS intensity. These observations confirm that the use of Au resulted in a higher surface area, providing amplification of the hot spots. Therefore, such a simple approach of the controlled decoration of Au over GO could successfully enhance the SERS activity. The peak intensities at 1076 cm^−1^ and 1586 cm^−1^ were chosen to calculate the SERS enhancement factor for the Au decorated GO sheet. The SERS enhancement factor is evaluated by comparing the SERS signals obtained from the 4-MBA capped structures to the Raman intensities acquired from the bulk 4-MBA molecule using the formula:

Enhancement factor (EF) = I_SERS_/I_NR_ × C_NR_/C_SERS_ (1)

where, I_SERS_ and I_NR_ correspond to the calculated intensity of the SERS spectra of the molecule which is adsorbed on the nano-hybrid surface and the normal Raman spectrum of the bulk molecule, respectively. C_NR_ and C_SERS_ are the corresponding 4-MBA concentrations for the normal Raman and SERS substrates [40]. We have expected that the 4-MBA molecules are evenly distributed on the SERS substrate. So, 10 μL of the 4-MBA solution having a 10^−7^ M concentration was pipetted and casted on the SERS substrate and the droplet evaporated in air for the experiment.

The calculated SERS enhancement factor (EF) for the Au-GO@13.2 Jcm^−2^ at 1076 cm^−1^ is 7.9 × 10^6^, respectively. Therefore, such nano-hybrids demonstrate higher SERS efficiency than the bare Au NPs and their excellent stability is in very good agreement with the literature [41].

Moreover, it should be noted that the Au nanoparticles with small dimensions are distributed throughout the GO surface thereby creating ‘hot spots’. Figure 7b represents the SERS spectra at different concentrations of 4-MBA, i.e., peaks from 10^−7^ M to 10^−12^ M were adsorbed on the surface of Au-GO@13.2 Jcm^−2^ sample. From Figure 7b, it is evident that the peaks corresponding to 4-MBA can be sensed even at a very low concentration of 4-MBA at 10^−12^ M thereby indicating a very high sensitivity of SERS substrate. Figure 7c represents the SERS Raman mapping image of the Au-GO@13.2 Jcm^−2^ sample, in which the red region corresponds to the ‘hot spots’. The Au-GO nano-hybrids are believed to be a favourable candidate for the SERS applications due to their surface roughness, which in turn provides a higher surface area for the more adsorption of the SERS-active molecules. The huge enhancement of the SERS signals from the Au-GO nano-hybrids is attributed to the presence of ultra-pure Au NPs, which serves as ‘hot spots’ during the SERS analysis and results in the strong coupling between the surface plasmons of Au NPs thereby enhancing the local electromagnetic field [42]. In the case of our samples, Au nanostructures are randomly distributed over the surface of the GO sheets. With improved structural design and enhanced SERS activity, these nano-hybrids could act as a new type of plasmonic nanomaterial with great potential for various applications including detection of trace chemicals, biomolecules, and in the detection of pathogens for food safety. Hence, the Au decorated GO could be excellent substrates for SERS due to their tunable plasmonic properties, more accessible surface area and chemical stability.

### 3.5. Au-GO Nano-Hybrids for the Catalytic Reduction of 4-nitrophenol

The catalytic reduction of 4-nitrophenol (4-NP) to 4-aminophenol (4-AP) by NaBH_4_ was performed as a time-controlled reaction in order to establish the catalytic activity of the Au-GO nano-hybrids. For that, 2.2 mL of water was mixed with 30 μL of 4-NP at a 10 mM concentration in a quartz cuvette at room temperature. To this solution, when 200 μL of 0.1 M, the NaBH_4_ solution was added. There was an instant change in the solution colour from light yellow to dark yellow. To this mixture, 0.5 mL of bare Au NPs and Au-GO nano-hybrid samples were added and the UV-Vis absorption spectra of the samples were recorded at certain specific intervals in the range of 200–800 nm at room temperature.

**Figure 8:**
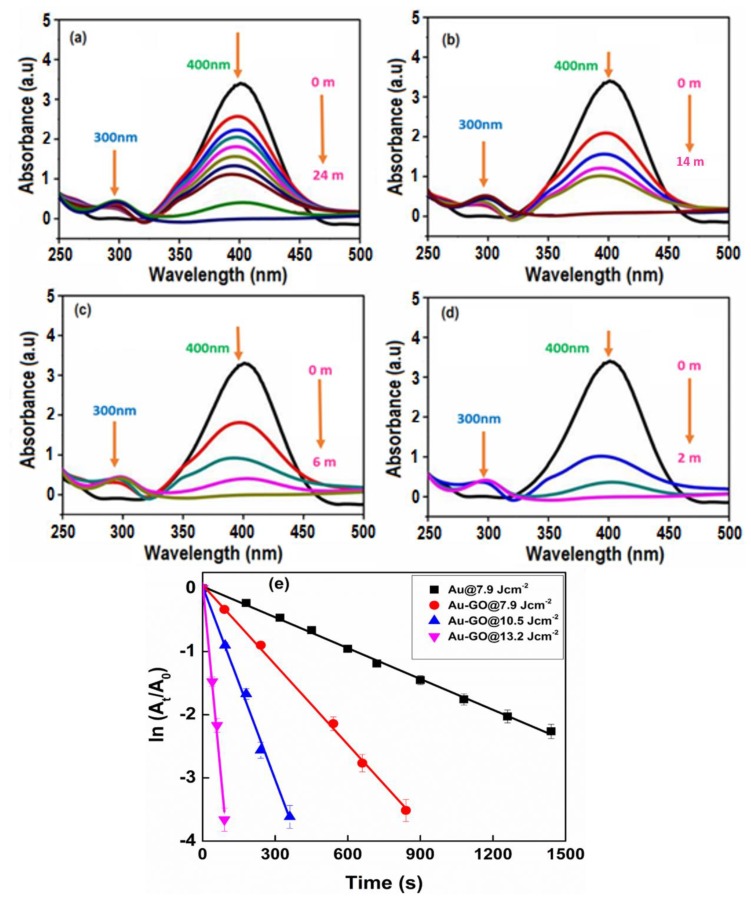
Time-resolved UV-Vis absorption spectra attributed to the catalytic reduction of 4-nitrophenol (4-NP) to 4-aminophenol (4-AP) with (**a**) Au NPs)@7.9 Jcm^−2^; (**b**) Au-GO@7.9 Jcm^−2^; (**c**) Au-GO@10.5 Jcm^−2^; (**d**) Au-GO@13.2 Jcm^−2^; (**e**) plot of ln(*At/A_0_*) against the reaction time of various proportions of the Au-GO samples.

For a quantitative investigation of the catalytic activity of the Au NPs and Au decorated GO sheets (Au-GO nano-hybrids), the reduction of 4-nitrophenol (4-NP) to 4-aminophenol (4-AP) by NaBH_4_ was chosen as a standard at room temperature [43]. The catalytic reduction reaction can easily be demonstrated by the UV-Vis absorption spectroscopy. The absorption peak of 4-NP is shifted to a higher wavelength (400 nm), upon the addition of NaBH_4_ due to the formation of an intermediate compound 4-nitrophenolate. In the absence of the as-prepared catalyst (Au-GO nano-hybrids), the absorption peak of 4-nitrophenolate at 400 nm remains unaltered over several times thereby confirming that the reduction reaction did not take place. Figure 8a–d represents the time-dependent UV-Vis absorption spectra corresponding to the catalytic reduction of 4-nitrophenol to 4-aminophenol by the Au and Au-GO nano-hybrids at different laser energies, respectively. In the absence of the catalyst (Au-GO nano-hybrid), the 400 nm absorption peak (due to 4-nitrophenolate) remains constant. However, after the small addition of the Au-GO samples, the absorption peak intensity at 400 nm slowly decreased and a new peak centered at 300 nm emerged corresponding to the 4-aminophenol and the colour of the solution turned transparent from the previous dark yellow. Herein, the nano-hybrid acted as a catalyst promoting the electrons transfer from BH_4_^−^ to 4-NP, facilitating the reduction process.

The time required for the completion of the reaction varied with the different Au-GO hybrids and Au itself. With the use of the Au-GO@13.2 Jcm^−2^ laser fluence, the reduction reaction was completed within 2 min as shown in Figure 8d and was found quicker than earlier reports. It should be noted that upon increasing the concentration of Au, the reaction time essential for the reduction was shortened. Additionally, the concentration of NaBH_4_ extensively goes beyond that of 4-NP, the reduction rate was found to be independent of the NaBH_4_ concentration. Therefore, the first order kinetics approximation may be suitable for calculating the apparent rate constants (*k_app_*) [44,45]. The reduction rate constant can be calculated according to pseudo first-order kinetics,

ln*(C_t_/C_o_)* = ln*(A_t_/A_o_) = k_app_ t* (2)

where *k_app_* corresponds to the apparent rate constant, and A_t_ and A_o_ represents the absorption of 4-NP at time *t* and its initial time, respectively. Figure 8e represents the relationship between ln (*A_t_/A_o_*) and the reaction time *t*, which exactly match with the first order reaction kinetics. Each sample exhibits a good linear fit of ln(*A_t_/A_o_*) with the reaction time. The *k_app_* values were obtained from the slope of the plot and the details are listed in Table 1. For Au-GO@13.2 Jcm^−2^ [*k_app_* = 40.2 × 10^−3^ s^−1^], Au-GO@10.5 Jcm^−2^ [*k_app_* = 10.2 × 10^−3^ s^−1^), Au-GO@7.9 Jcm^−2^ [*k_app_* = 4.2 × 10^−3^ s^−1^] and for Au@ 7.9 Jcm^−2^ [*k_app_* = 1.6 × 10^−3^ s^−1^)]. From the results, it is evident that there is a significant increase in the *k_app_* value of the nano-hybrids synthesised at higher laser fluences. The *k_app_* value for the sample Au-GO@13.2 Jcm^−2^ is found to be 40.2 × 10^−3^ s^−1^, which is significantly higher than the other two samples and AuNPs itself. Hence, the catalytic activity of the Au-GO nano-hybrids obtained at higher laser fluences was found to be greater than that of the bare Au NPs.

Moreover, the catalytic reduction activity of Au-GO nano-hybrid with other materials has also been compared and the details are summarized in Table 2.

## 4. Conclusions

In this work, we establish the benefits of using a laser-assisted synthesis protocol for the production of highly pure, ligand-free gold nanoparticles that are directly anchored on to the surface of the graphene oxide matrix. The tuning of laser parameters influences the size and concentration of the AuNPs. There is not much considerable alteration in the properties of graphene oxide after laser ablation except for a modest increase in the GO reduction. Throughout the laser ablation process for the in situ generation of gold nanoparticles on the GO matrix, the inherent properties of GO were observed to be unaltered. Here, the graphene oxide is functioning as a capping agent that controls the size of the Au nanoparticles. We further demonstrated that the Au-GO hybrid material exhibits outstanding catalytic and SERS performance when compared with the metal nanoparticles alone. Both the synthesis protocol by laser ablation and the applications of the designed nano-hybrid provides motivations for future advancement in the materials science platform, especially in the catalysis and chemical sensing.

## Figures and Tables

**Table 1:** Catalytic reduction time and *k_app_* of AuNPs and Au-GO nano-hybrids.

Product	Au@7.9 Jcm^−2^	Au-GO@7.9 Jcm^−2^	Au-GO@10.5 Jcm^−2^	Au-GO@13.2 Jcm^−2^
Reduction time (in seconds)	1440	840	360	120
*k*_app_ (10^−3^ s^−1^)	1.6	4.2	10.2	40.2

**Table 2:** Comparison of the apparent rate constants *k*_app_ of different catalysts for the reduction of 4-nitrophenol.

Samples	Apparent Rate Constant *k_app_,* (10^−3^ s^−1^)	Reference
Au-GO@13.2 Jcm^−2^ via PLA	40.2	This work
Au-GO@10.5 Jcm^−2^ via PLA	10.2	This work
Au-GO@7.9 Jcm^−2^ via PLA	4.2	This work
Au@7.9 Jcm^−2^ via PLA	1.6	This work
rGO-AgAu bimetallic nanocomposite via green synthesis	1.4	[46]
Au/graphene hydrogel via chemical reduction	3.1	[47]
Ag-Pt NWs via chemical reduction	6.9	[48]
Ag/Au bimetallic nanostructures via chemical reduction	6.1	[49]
Ag–Au-C composite via chemical reduction	1.6	[50]
